# Spontaneous formation of neutrophil extracellular traps in serum‐free culture conditions

**DOI:** 10.1002/2211-5463.12222

**Published:** 2017-05-02

**Authors:** Go Kamoshida, Takane Kikuchi‐Ueda, Satoshi Nishida, Shigeru Tansho‐Nagakawa, Hirotoshi Kikuchi, Tsuneyuki Ubagai, Yasuo Ono

**Affiliations:** ^1^ Department of Microbiology and ImmunologyTeikyo University School of MedicineTokyoJapan

**Keywords:** neutrophil, neutrophil extracellular traps, serum

## Abstract

Neutrophils play a critical role in the innate immune response. Recently, a new neutrophilic biological defense mechanism, termed neutrophil extracellular traps (NETs), has been attracting attention. Neutrophils have been observed to release both lysosomal enzymes and their nuclear contents, including unfolded chromatin, which together trap and inactivate bacteria. The environment in tissues where neutrophils act is thought to be different from that of the blood serum. In this study, we assessed the effect of serum on NET formation. We found that neutrophils spontaneously form NETs in serum‐free cultivation conditions at early times. These NETs functioned properly to trap bacteria. Furthermore, we demonstrated that reactive oxygen species play a critical role in the spontaneous formation of NETs. These results suggest that the serum condition must be considered in studies on neutrophils, including the formation and mechanism of action of NETs.

**Abbreviations**
BSA: bovine serum albumin
CFU: colony‐forming units
DAPI: 4′,6‐diamidino‐2‐phenylindole
DMEM: Dulbecco's modified Eagle's medium
FBS: fetal bovine serum
HBSS: Hanks’ balanced salt solution
MOI: multiplicity of infection
NAC: *N*‐acetyl‐l‐cysteine
NET: neutrophil extracellular trap
PMA: phorbol 12‐myristate 13‐acetate
ROS: reactive oxygen species

Neutrophils are immune cells that play a pivotal role during the initial immune response to various bacterial infections. They migrate towards the site of infection and act against bacteria by phagocytosis and producing toxic factors such as reactive oxygen species (ROS) 1, 2. Additionally, neutrophils were shown to release their nuclear contents, including unfolded chromatins and lysosomal enzymes such as neutrophil elastase, which play a critical role in bacterial killing by trapping and inactivating the bacteria 3, 4, 5, 6, 7. This biological defense mechanism of the neutrophils is termed neutrophil extracellular traps (NETs). This mechanism has gained considerable attention and is being examined worldwide. Furthermore, NETs have been considered as a novel cell death mechanism by neutrophils and the associated process has been termed NETosis to distinguish it from necrosis and apoptosis 8. Although NETs are effective for protection against bacterial infection, they also induce inflammation, autoimmune diseases and thrombosis by releasing several endogenous intracellular factors. Therefore, NETs appear to have paradoxical functions 9, 10. NET formation is a newly characterized cellular event of the neutrophils, and the precise functions of NETs and the molecular mechanisms of their induction remain to be elucidated.

Previous investigations described several inducers of NET formation. Phorbol 12‐myristate 13‐acetate (PMA) and various bacteria were shown to induce NETs, and these stimulations have been used to investigate the functions and detailed molecular mechanisms of NET formation 5, 7, 11. However, many of these studies were performed *in vitro* owing to challenges associated with *in vivo* observations. In the *in vitro* experimental system, neutrophils purified from blood are often utilized; however, a consensus on experimental conditions, such as the origin of neutrophils, their purification method, length and method of preservation following purification, and cultivation conditions including serum concentration and batch‐to‐batch variation, has not been reached and these conditions often vary between studies. *In vivo*, neutrophils are typically present in the serum‐containing blood; thus, the presence of serum during culture is thought to be important for *in vitro* studies. Since the serum condition in the tissue where neutrophils act to fight bacteria might be different from that of the blood, i.e. serum concentration in the tissue is typically lower than that in blood, serum condition may affect the function of neutrophils. Nevertheless, serum conditions are often not described in previous reports. In the present study, neutrophils were cultured in serum‐containing or serum‐free cultivation conditions to evaluate the effect of serum on NET formation. We discovered that neutrophils can spontaneously form a NET‐like structure in serum‐free cultivation conditions. Furthermore, we provided evidence that ROS played a pivotal role during the spontaneous formation of NETs induced by serum‐free culture conditions.

## Materials and methods

### Preparation of human neutrophils and serum

Neutrophils were isolated from the peripheral blood of healthy volunteers (*n* = 10), as described previously 12. Heparin‐treated blood was mixed with 1% dextran (*M*_r_ 200 000) in saline to sediment most of the erythrocytes. Thereafter, samples were incubated for 30 min and the supernatant was subsequently subjected to Lymphosepar I (Immuno‐Biological Laboratories, Takasaki, Japan) density gradient centrifugation at 500 ***g*** for 30 min. Neutrophils were purified from pelleted cells by hypotonic lysis of the remaining erythrocytes. Purity of the neutrophils was greater than 95%. Purified neutrophils were used within 30 min.

Human serum was prepared from the peripheral blood of healthy volunteers (*n* = 5). Heparin‐treated human blood was incubated for 1 h at 4 °C. Following centrifugation at 500 ***g*** for 10 min, the serum supernatants were collected. After confirming that there was no significant batch‐to‐batch variation between serum preparations, the same serum batch was used for all experiments.

Written informed consent was obtained from all study participants. The study was approved by the Ethical Review Committee of Teikyo University School of Medicine.

### Visualization of NETs

Neutrophils (1 × 10^6^ cells·mL^−1^) were cultured on non‐coated glass slides (1‐9646‐12; As One, Osaka, Japan) for 1–5 h in RPMI 1640 (Sigma‐Aldrich, St. Louis, MO, USA) containing 2% human serum or in a serum‐free condition at 37 °C with 5% CO_2_. Cells were fixed with 5% formaldehyde at room temperature for 10 min and washed three times with PBS. DNA was stained with 4′,6‐diamidino‐2‐phenylindole (DAPI; SouthernBiotech, Birmingham, AL, USA) and visualized with a fluorescence microscope (BX53; Olympus, Tokyo, Japan). Immunostaining of neutrophil elastase was performed as previously described 13. Briefly, neutrophils were cultured for 1 h and fixed as described above. Fixed cells were incubated with an anti‐neutrophil elastase antibody (Abcam, Cambridge, MA, USA; 1 : 200 dilution) and subsequently with an Alexa Fluor 555‐conjugated anti‐rabbit IgG (Cell Signaling Technology, Danvers, MA, USA; 1 : 1000 dilution). Following each incubation step, cells were washed five times with PBS. Stained cells were visualized with fluorescence microscopy as described above.

### Quantification of NETs

Extracellular DNA was stained with 5 μm SYTOX Green (Life Technologies, Carlsbad, CA, USA), a fluorescent membrane‐impermeable DNA dye. Fluorescence was quantified using a microplate reader equipped with filters to detect excitation/emission maxima of 504/523 nm (EnSpire; PerkinElmer, Waltham, MA, USA). When indicated, neutrophils were stimulated with 20 or 200 nm PMA, or cultured in the presence of 100 U·mL^−1^ DNase I (TaKaRa, Osaka, Japan), 40 mg·mL^−1^ bovine serum albumin (BSA; Wako Pure Chemical Industries, Osaka, Japan), 40 mg·mL^−1^ human serum albumin (Wako), heat‐treated (complement‐inactivated) serum, protein G/A sepharose‐treated (immunoglobulin‐depleted) serum, 10 mm EDTA, or 5 or 20 mm *N*‐acetyl‐l‐cysteine (NAC; Wako).

### Bacteria‐trapping assay

Neutrophils (1 × 10^6^ cells·mL^−1^) were cultured for 1 h in a serum‐free condition to induce the formation of NETs. *Escherichia coli* cells (ATCC25922; ATCC, Manassas, VA, USA) were labeled with PKH67 green fluorescence (Sigma‐Aldrich), added to NETs induced by the serum‐free culture condition at a multiplicity of infection (MOI) of 50, and cultured for 15 min. Cells were then washed five times with PBS and fixed with 5% formaldehyde. DNA was stained with DAPI and cells were visualized by fluorescence microscopy, as described above.

### NET bacteria‐killing assay

Neutrophils (1 × 10^6^ cells·mL^−1^) were cultured for 1 h in a serum‐free condition to induce the formation of NETs. Cytochalasin B (5 μg·mL^−1^; Wako) was then added and the cells were incubated further for 15 min. Subsequently, *E. coli* was added into the well at an MOI of 50 with or without DNase I (100 U·mL^−1^), and the cells were cultured for 1 h. Appropriate dilutions of the culture supernatants were spread onto LB agar plates and the plates were incubated at 37 °C overnight. Colonies were counted to determine the colony‐forming units (CFU) of the surviving bacteria.

### Detection of reactive oxygen species

To visualize intracellular ROS with fluorescence microscopy, we stained neutrophils with CellROX Deep Red Reagent (Life Technologies). In certain experiments, neutrophils were cultured in the presence of NAC (5 or 20 mm), as indicated.

### Statistical analysis

Data are expressed as the mean ± standard deviation (SD). Results were compared by paired Student's *t* test and differences at *P* values of < 0.01 were considered statistically significant.

## Results and Discussion

### Neutrophils formed spontaneous NETs in serum‐free culture conditions

**Figure 1:**
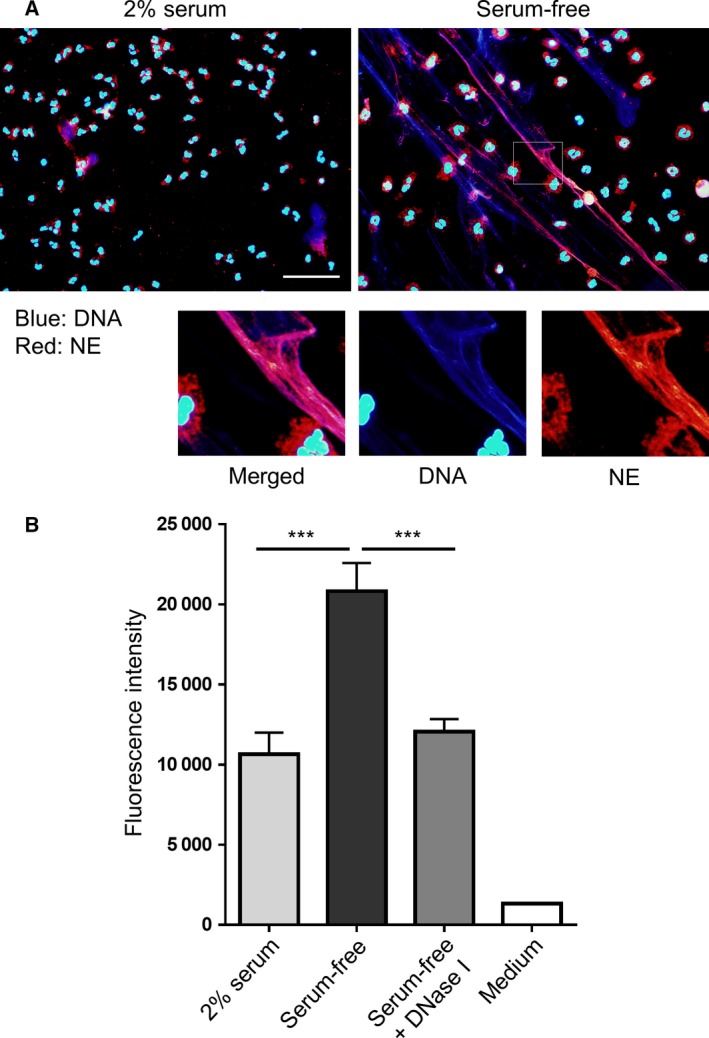
Neutrophils form NETs after cultivation in a serum‐free condition for 1 h. Neutrophils were cultured for 1 h in RPMI 1640 in the presence of 2% human serum or in a serum‐free condition. (A) Fixed neutrophils were stained with anti‐neutrophil elastase antibody (NE; red). DNA was stained with DAPI (blue). Stained cells were visualized with fluorescence microscopy. Scale bar = 50 μm. (B) Extracellular DNA was stained with SYTOX Green and fluorescence was quantified using a microplate reader. Where indicated, neutrophils were cultured in the presence of DNase I. Data are shown as the mean ± SD;* n* ≥ 3 per group. ***P < 0.001 relative to the serum‐free condition. Results are representative of three or more independent experiments (using neutrophils from different donors).

**Figure 2:**
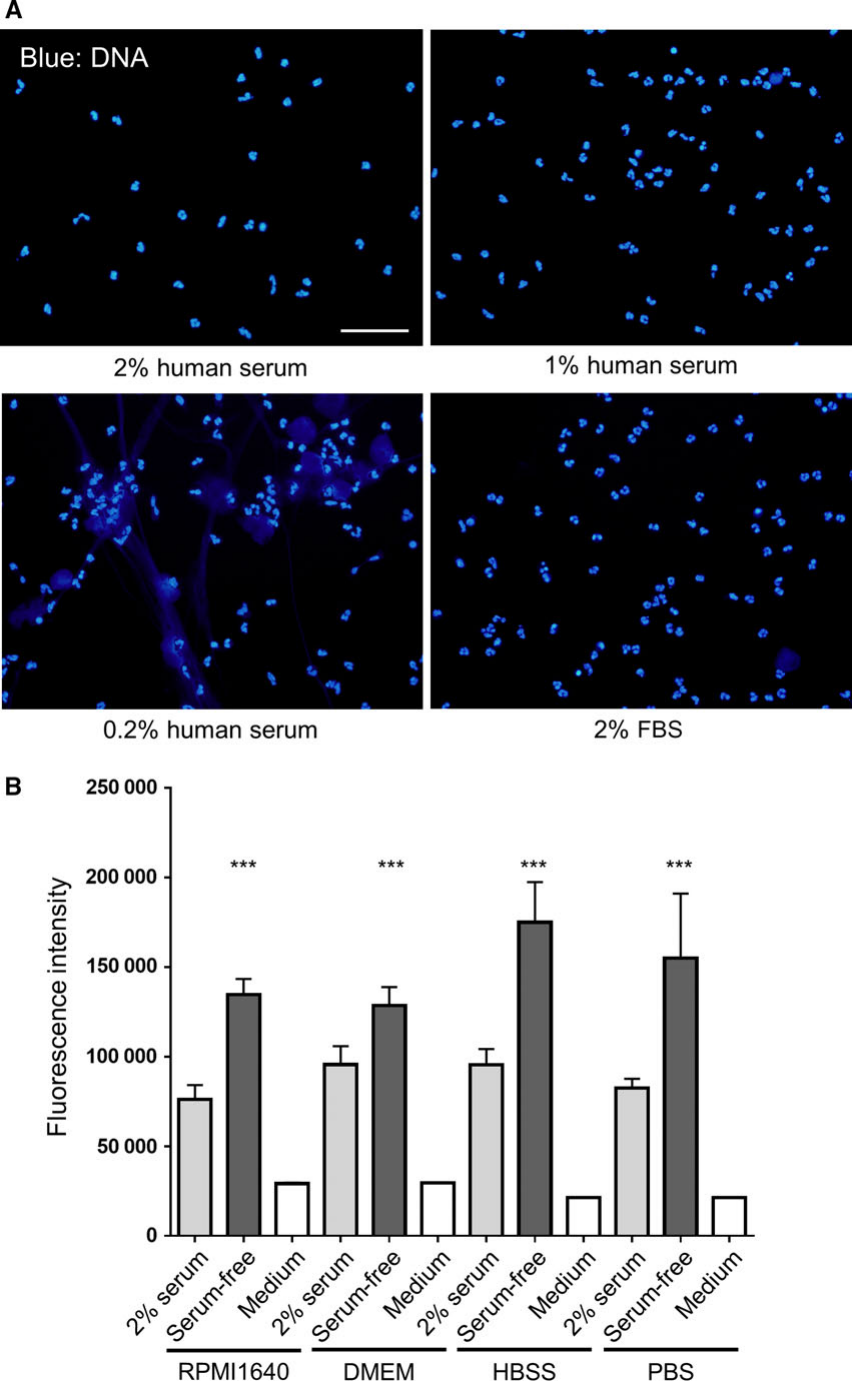
Effect of serum concentration and medium or buffer on the formation of NETs. (A) Neutrophils were cultured for 1 h in RPMI 1640 containing 2, 1, 0.2% human serum, or 2% FBS. Cells were fixed and stained with DAPI (blue) and observed under a fluorescence microscope. Scale bar = 50 μm. (B) Neutrophils were cultured for 1 h in RPMI 1640, DMEM, HBSS, or PBS in the presence of 2% human serum or in a serum‐free condition. Extracellular DNA was stained with SYTOX Green and fluorescence was quantified using a microplate reader. Data are shown as the mean ± SD;* n* ≥ 3 per group. ***P < 0.001 relative to the serum‐free condition. Results are representative of three or more independent experiments (using neutrophils from different donors).

To assess NET formation, neutrophils were cultured for 1 h in serum‐free RPMI 1640, and stained with DAPI and anti‐neutrophil elastase antibody. We observed disruption and release of nuclear DNA into the extracellular space, which resembled NETs. Neutrophil elastase was found to co‐localize with extracellular DNA in a serum‐free condition (Fig. 1A). Nevertheless, the distinctive shape of neutrophil nuclei was also observed. In contrast, when neutrophils were cultured in media containing 2% human serum, nearly all neutrophils displayed nuclear DNA. To quantify the amount of extracellular DNA, we measured SYTOX Green‐stained DNA in the medium. The amount of extracellular DNA was increased in the serum‐free culture condition when compared with that observed in the serum‐containing culture condition. The extracellular DNA induced by the serum‐free condition was lowered to the control level (2% serum) by DNase I treatment (Fig. 1B). These results demonstrated that spontaneous NET formation was induced when neutrophils were cultured for 1 h in a serum‐free condition. Although a NET‐like structure was not observed when 1% human serum or 2% fetal bovine serum (FBS) was used, it was apparent in the 0.2% human serum culture condition (Fig. 2A). The spontaneous formation of NETs in serum‐free conditions was also observed when Dulbecco's modified Eagle's medium (DMEM), Hanks’ balanced salt solution (HBSS), or PBS was used instead of RPMI 1640 (Fig. 2B and Fig. S1).

**Figure 3:**
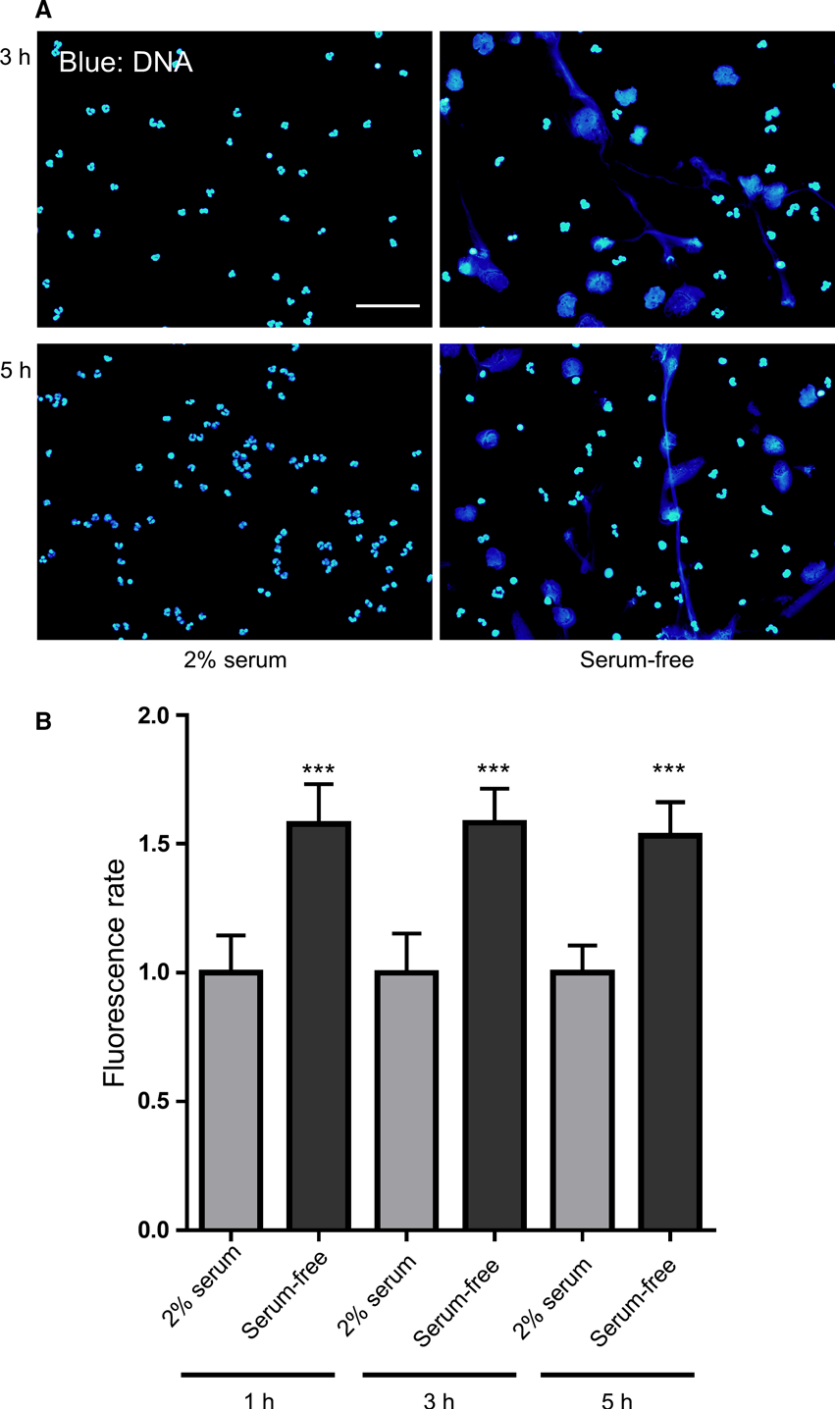
Kinetics of NET formation induced by serum‐free culture conditions. (A) Neutrophils were cultured for 3 or 5 h in RPMI 1640 containing 2% human serum or in a serum‐free condition. Cells were fixed and stained with DAPI (blue) and visualized under a fluorescence microscope. Scale bar = 50 μm. (B) Extracellular DNA was stained with SYTOX Green at 1, 3 and 5 h of culture and fluorescence was quantified using a microplate reader. The fluorescence rate was expressed relative to neutrophils in 2% serum at the corresponding time point (set as 1.0). Data are shown as the mean ± SD;* n* ≥ 3 per group. ***P < 0.001 relative to neutrophils in 2% serum. Results are representative of three or more independent experiments (using neutrophils from different donors).

Further, to assess the kinetics of serum‐free induction of NETs, a 5‐h neutrophil culture was visualized under a fluorescence microscope (Fig. 3A) and by time‐lapse microscopy (Movies S1 and S2). NETs derived from extracellular DNA were visible at 1–5 h of culture. Additionally, we measured the amount of extracellular DNA to quantify NET formation. We found that the amount of extracellular DNA was increased at 1, 3 and 5 h of culture in a serum‐free condition relative to that observed in the presence of 2% serum. However, the extracellular DNA was not increased in a time‐dependent manner (Fig. 3B). These observations indicated that neutrophils can form NETs in serum‐free conditions early during culture, i.e. at 1–5 h of culture.

### Kinetics, magnitude and functionality of NETs induced by a serum‐free culture condition

**Figure 4:**
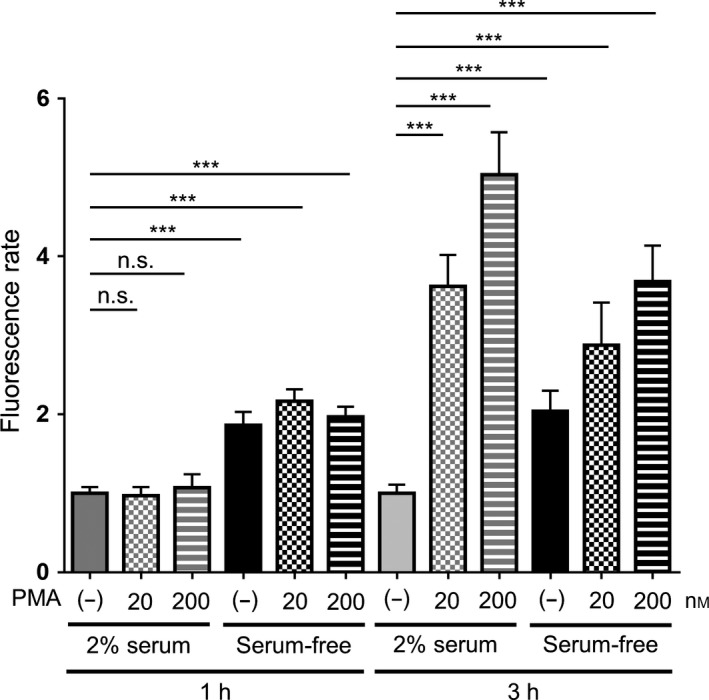
Comparison of NET formation induced by serum‐free conditions or PMA. Neutrophils were cultured for 1 or 3 h in RPMI 1640 containing 2% human serum, serum‐free, 2% serum with PMA stimulation, or serum‐free with PMA stimulation. Extracellular DNA was stained with SYTOX Green and fluorescence was quantified using a microplate reader. The fluorescence rate was expressed relative to neutrophils in 2% serum at the corresponding time point (set as 1.0). Data are shown as the mean ± SD;* n* ≥ 3 per group. ***P < 0.001. Results are representative of three or more independent experiments (using neutrophils from different donors).

PMA has been used as a positive control for NET induction in most studies of NETs 3, 14. Therefore, we compared NET formation induced by a serum‐free culture condition with those induced by PMA. Neutrophils cultured in a serum‐free condition displayed increased extracellular DNA at 1 and 3 h of culture. However, 1‐h PMA treatment failed to increase the extracellular DNA level. Nevertheless, at 3 h of culture, the amount of extracellular DNA was significantly higher in PMA‐induced neutrophils than that observed under a serum‐free cultivation (Fig. 4). Thus, the spontaneous formation of NETs induced by serum‐free conditions took place earlier than those induced by PMA, although lower in magnitude. In addition, PMA induction in serum‐free culture did not result in earlier or stronger NET inductions when compared with that observed in serum‐free conditions without PMA.

**Figure 5:**
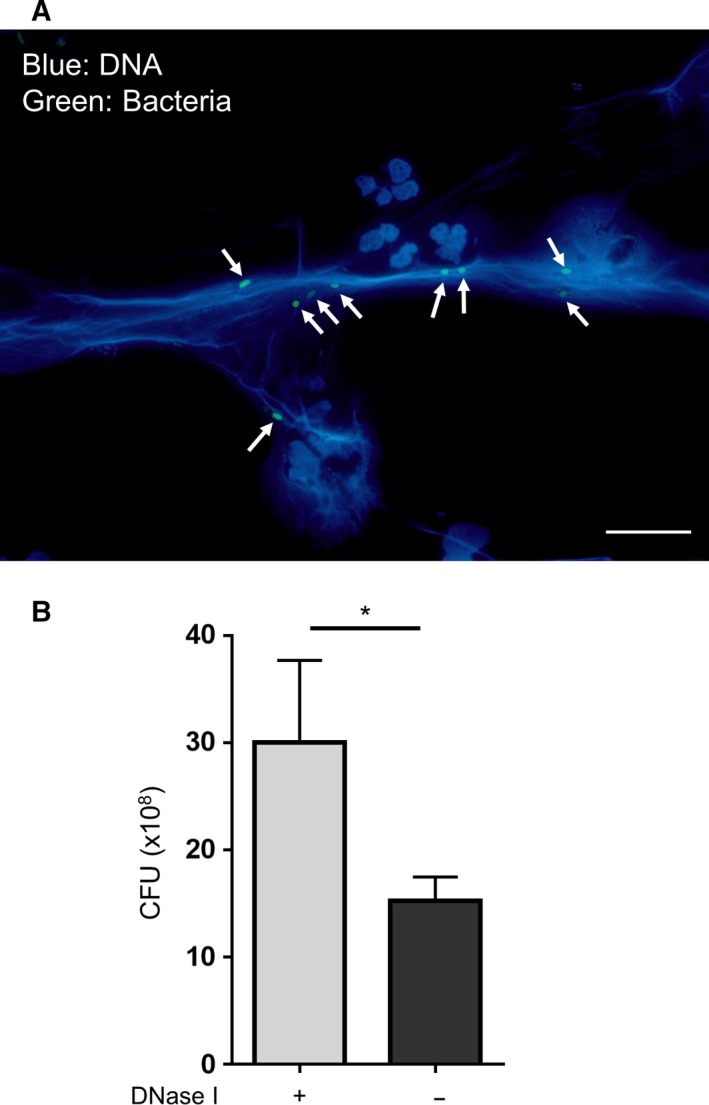
Functionality of NETs induced by serum‐free culture conditions. (A) Neutrophils were cultured for 1 h in a serum‐free condition to induce the formation of NETs. *Escherichia coli* (green) was added to the NETs and cultured for 15 min. After washing, cells were fixed and stained with DAPI (blue). Cells were observed under a fluorescence microscope. Arrows indicate *E. coli* trapped in NETs. Scale bar = 20 μm. (B) Neutrophils were cultured for 1 h in a serum‐free condition to induce NET formation. *E. coli* was added to the well and cultured for 1 h. The culture supernatants were spread onto LB agar plates and colonies were counted to determine the CFU of surviving bacteria. Data are shown as the mean ± SD;* n* ≥ 3 per group. *P < 0.05. Results are representative of three or more independent experiments (using neutrophils from different donors).

To analyze the functionality of NETs induced by a serum‐free condition, we performed a bacteria‐trapping assay. Neutrophils were cultured for 1 h in a serum‐free condition to induce spontaneous formation of NETs, and fluorescence‐labeled *E. coli* was added to the NETs. *E. coli* was found to be trapped in NETs formed in serum‐free conditions (Fig. 5A). Further, we carried out an *in vitro* NET bacteria killing assay to investigate the capacity of these NETs to kill bacteria. The results showed that bacteria trapped in NETs were indeed killed (Fig. 5B), demonstrating that the spontaneously formed NETs in serum‐free culture conditions were functioning effectively as a trap for bacteria. Although a significant number of neutrophils are found in the blood, neutrophils are also present in tissues to fight foreign materials such as bacteria. However, serum concentration in the tissue is thought to be lower than that in the blood. In the present study, we found that serum‐free culture of neutrophils promoted the spontaneous formation of NETs. Results of this study suggest that a low level or absence of serum proteins in tissues may aid the formation of NETs by neutrophils to counter bacteria at the site of infection.

### Mechanism of spontaneous NET formation in serum‐free culture

**Figure 6:**
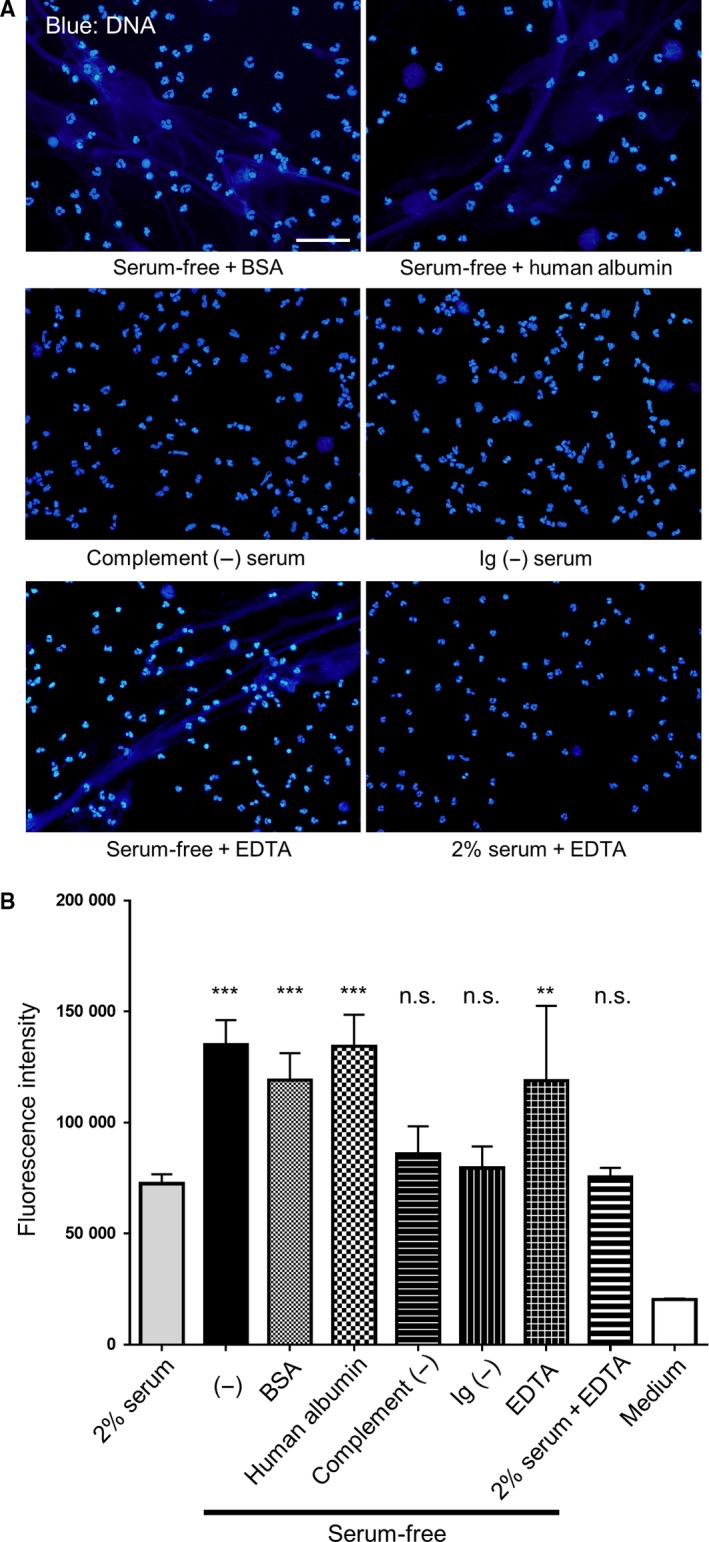
Effect of serum components on the formation of NETs. Neutrophils were cultured for 1 h in RPMI 1640 containing 40 mg·mL^−1^ BSA, 40 mg·mL^−1^ human serum albumin, 2% complement‐inactivated serum (by heat treatment; Complement (−)), 2% immunoglobulin‐depleted serum (by protein G/A sepharose; Ig (−)), or EDTA (10 mm) in the presence or absence of serum. (A) Cells were fixed and stained with DAPI (blue) and visualized under a fluorescence microscope. Scale bar = 50 μm. (B) Extracellular DNA was stained with SYTOX Green and fluorescence was quantified using a microplate reader. Data are shown as the mean ± SD;* n* ≥ 3 per group. **P < 0.01, ***P < 0.001 relative to the serum‐free condition. Results are representative of three or more independent experiments (using neutrophils from different donors).

Lastly, we aimed to determine the mechanism of serum‐free spontaneous induction of NETs. First, we examined the effect of albumin, which is often found in serum, on NET formation. We found that neither bovine nor human serum albumin reduced the formation of NETs. Furthermore, complement molecules and immunoglobulins in serum did not appear to affect NET formation. Several studies showed that serum may degrade NETs 15, 16. However, spontaneous formation of NETs was observed upon the inhibition of DNase activity with EDTA in serum‐free conditions, but not in the presence of 2% serum (Fig. 6). Thus, albumin, complements, immunoglobulins, and DNase did not appear to be involved in the serum‐free spontaneous formation of NETs.

**Figure 7:**
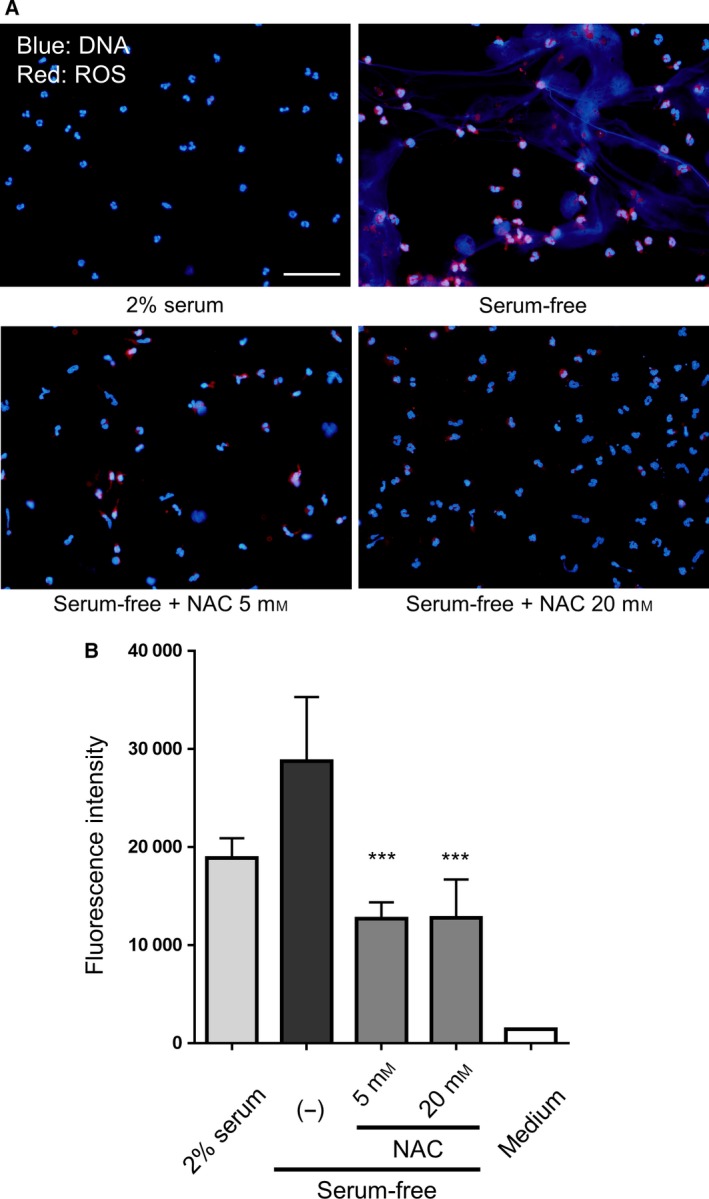
Effect of ROS on the formation of NETs induced by serum‐free culture conditions. Neutrophils were cultured for 1 h in RPMI 1640 containing 2% human serum or in a serum‐free condition. Where indicated, neutrophils were cultured in the presence of 5 or 20 mm NAC. (A) Cells were stained with CellROX Deep Red Reagent (red) and DAPI (blue), and visualized under a fluorescence microscope. Scale bar = 50 μm. (B) Extracellular DNA was stained with SYTOX Green and fluorescence was quantified using a microplate reader. Data are shown as the mean ± SD;* n* ≥ 3 per group. ***P < 0.001 relative to serum‐free cultivation (−). Results are representative of three or more independent experiments (using neutrophils from different donors).

Previous investigations have identified a critical role of ROS for NET formation 17, 18. Neutrophils of patients with chronic granulomatous diseases displayed impaired ROS production and exhibited little to no NET formation, suggesting a role of ROS in the induction of NETs 19. To specifically determine the effect of ROS on NET formation induced by serum‐free conditions, we assessed the formation of NETs following ROS inhibition by NAC. Both ROS production and NET formation were induced by serum‐free conditions, as observed by fluorescence microscopy. These inductions were concurrently suppressed by 5 or 20 mm of NAC (Fig. 7A). Additionally, we measured the amount of extracellular DNA to quantify the NET formation. We found that the release of extracellular DNA during serum‐free culture conditions was also suppressed by NAC treatment (Fig. 7B). These results suggested that ROS played an important role in the spontaneous formation of NETs induced by serum‐free culture conditions. A previous report showed that serum restricts the formation of NETs induced by PMA stimulation of more than 3 h, owing to its antioxidant activity 19; however, the antioxidant components in serum were not identified in the study. Another study demonstrated the ability of cold physical plasma to induce the formation of NETs 20. Cold physical plasma is an ionized gas with various components, including ROS. Thus, these findings suggested that both serum condition and ROS are important factors for NET formation. The identity and precise mechanism of action of the inhibitory factors in serum will need to be addressed in future studies.

In previous studies, PMA stimulation and bacteria were used to induce NET formation 11. However, subsequent analyses were challenging or inconceivable because PMA and bacteria often induce excessively robust signaling. In serum‐free culture conditions, the magnitude of NET induction was relatively small, which should permit subsequent analyses. Thus, NET induction by serum‐free stimulation may be useful to investigate the functions and molecular mechanisms of NETs. During NET formation, several endogenous factors, including intracellular DNA, are released; thus, the effects of these factors will need to be considered 10, 11, 21. Therefore, a consideration for serum culture conditions should not be limited to studies of NETs alone, but expanded to all *in vitro* studies of neutrophils.

## Conclusions

Our results demonstrated that serum‐free culture of neutrophils promoted the spontaneous formation of NETs, which was dependent on ROS generation. The results suggested that a low level or absence of serum in tissues may assist neutrophils to form NETs, which are important for neutrophil actions against bacterial infection. Furthermore, the experimental system described in this study may aid future efforts to elucidate the role of NETs in the pathogenesis of various diseases, such as cancer metastasis, coagulation and autoimmune diseases.

## Author contributions

GK designed and performed the experiments, analyzed and discussed the data, and wrote the manuscript. TK‐U, SN, ST‐N, HK and TU performed a portion of the experiments and discussed the data. YO supervised the study.

## Supporting information


**Fig. S1.** Effect of medium or buffer on the serum‐free induction of neutrophil extracellular traps (NETs).

**Movie S1.** Time‐lapse observation of neutrophil extracellular trap (NET) formation in serum‐containing culture conditions (2% serum).

**Movie S2.** Time‐lapse observation of neutrophil extracellular trap (NET) formation in serum‐free culture conditions.

## References

[1] Nathan C (2006) Neutrophils and immunity: challenges and opportunities. Nat Rev Immunol 6, 173–182. 16498448 10.1038/nri1785

[2] Nauseef WM and Borregaard N (2014) Neutrophils at work. Nat Immunol 15, 602–611. 24940954 10.1038/ni.2921

[3] Brinkmann V , Reichard U , Goosmann C , Fauler B , Uhlemann Y , Weiss DS , Weinrauch Y and Zychlinsky A (2004) Neutrophil extracellular traps kill bacteria. Science 303, 1532–1535. 15001782 10.1126/science.1092385

[4] Mantovani A , Cassatella MA , Costantini C and Jaillon S (2011) Neutrophils in the activation and regulation of innate and adaptive immunity. Nat Rev Immunol 11, 519–531. 21785456 10.1038/nri3024

[5] Brinkmann V and Zychlinsky A (2007) Beneficial suicide: why neutrophils die to make NETs. Nat Rev Microbiol 5, 577–582. 17632569 10.1038/nrmicro1710

[6] Papayannopoulos V and Zychlinsky A (2009) NETs: a new strategy for using old weapons. Trends Immunol 30, 513–521. 19699684 10.1016/j.it.2009.07.011

[7] Brinkmann V and Zychlinsky A (2012) Neutrophil extracellular traps: is immunity the second function of chromatin? J Cell Biol 198, 773–783. 22945932 10.1083/jcb.201203170 PMC3432757

[8] Galluzzi L , Vitale I , Abrams JM , Alnemri ES , Baehrecke EH , Blagosklonny MV , Dawson TM , Dawson VL , El‐Deiry WS , Fulda S *et al* (2012) Molecular definitions of cell death subroutines: recommendations of the Nomenclature Committee on Cell Death 2012. Cell Death Differ 19, 107–120. 21760595 10.1038/cdd.2011.96 PMC3252826

[9] Fuchs TA , Brill A , Duerschmied D , Schatzberg D , Monestier M , Myers DD Jr , Wrobleski SK , Wakefield TW , Hartwig JH and Wagner DD (2010) Extracellular DNA traps promote thrombosis. Proc Natl Acad Sci USA 107, 15880–15885. 20798043 10.1073/pnas.1005743107 PMC2936604

[10] Kaplan MJ and Radic M (2012) Neutrophil extracellular traps: double‐edged swords of innate immunity. J Immunol 189, 2689–2695. 22956760 10.4049/jimmunol.1201719 PMC3439169

[11] Cheng OZ and Palaniyar N (2013) NET balancing: a problem in inflammatory lung diseases. Front Immunol 4, 1. 23355837 10.3389/fimmu.2013.00001 PMC3553399

[12] Kamoshida G , Tansho‐Nagakawa S , Kikuchi‐Ueda T , Nakano R , Hikosaka K , Nishida S , Ubagai T , Higashi S and Ono Y (2016) A novel bacterial transport mechanism of *Acinetobacter baumannii* via activated human neutrophils through interleukin‐8. J Leukoc Biol 100, 1405–1412. 27365529 10.1189/jlb.4AB0116-023RR

[13] Kamoshida G , Kikuchi‐Ueda T , Tansho‐Nagakawa S , Nakano R , Nakano A , Kikuchi H , Ubagai T and Ono Y (2015) *Acinetobacter baumannii* escape from neutrophil extracellular traps (NETs). J Infect Chemother 21, 43–49. 25287154 10.1016/j.jiac.2014.08.032

[14] Neeli I and Radic M (2013) Opposition between PKC isoforms regulates histone deimination and neutrophil extracellular chromatin release. Front Immunol 4, 38. 23430963 10.3389/fimmu.2013.00038 PMC3576869

[15] Leffler J , Gullstrand B , Jonsen A , Nilsson JA , Martin M , Blom AM and Bengtsson AA (2013) Degradation of neutrophil extracellular traps co‐varies with disease activity in patients with systemic lupus erythematosus. Arthritis Res Ther 15, R84. 23945056 10.1186/ar4264 PMC3978901

[16] Hakkim A , Furnrohr BG , Amann K , Laube B , Abed UA , Brinkmann V , Herrmann M , Voll RE and Zychlinsky A (2010) Impairment of neutrophil extracellular trap degradation is associated with lupus nephritis. Proc Natl Acad Sci USA 107, 9813–9818. 20439745 10.1073/pnas.0909927107 PMC2906830

[17] Yost CC , Cody MJ , Harris ES , Thornton NL , McInturff AM , Martinez ML , Chandler NB , Rodesch CK , Albertine KH , Petti CA *et al* (2009) Impaired neutrophil extracellular trap (NET) formation: a novel innate immune deficiency of human neonates. Blood 113, 6419–6427. 19221037 10.1182/blood-2008-07-171629 PMC2710935

[18] Remijsen Q , Kuijpers TW , Wirawan E , Lippens S , Vandenabeele P and Vanden Berghe T (2011) Dying for a cause: NETosis, mechanisms behind an antimicrobial cell death modality. Cell Death Differ 18, 581–588. 21293492 10.1038/cdd.2011.1 PMC3131909

[19] Fuchs TA , Abed U , Goosmann C , Hurwitz R , Schulze I , Wahn V , Weinrauch Y , Brinkmann V and Zychlinsky A (2007) Novel cell death program leads to neutrophil extracellular traps. J Cell Biol 176, 231–241. 17210947 10.1083/jcb.200606027 PMC2063942

[20] Bekeschus S , Winterbourn CC , Kolata J , Masur K , Hasse S , Broker BM and Parker HA (2016) Neutrophil extracellular trap formation is elicited in response to cold physical plasma. J Leukoc Biol 100, 791–799. 26992432 10.1189/jlb.3A0415-165RR

[21] Kambas K , Mitroulis I , Apostolidou E , Girod A , Chrysanthopoulou A , Pneumatikos I , Skendros P , Kourtzelis I , Koffa M , Kotsianidis I *et al* (2012) Autophagy mediates the delivery of thrombogenic tissue factor to neutrophil extracellular traps in human sepsis. PLoS One 7, e45427. 23029002 10.1371/journal.pone.0045427 PMC3446899

