# Supplementary figures and images for: Spontaneous formation of neutrophil extracellular traps in serum‐free culture conditions
Source: FEBS Open Bio. 2017 May 2;7(6):877–86. doi: 10.1002/2211-5463.12222 (PMC5458474; doi:10.1002/2211-5463.12222)

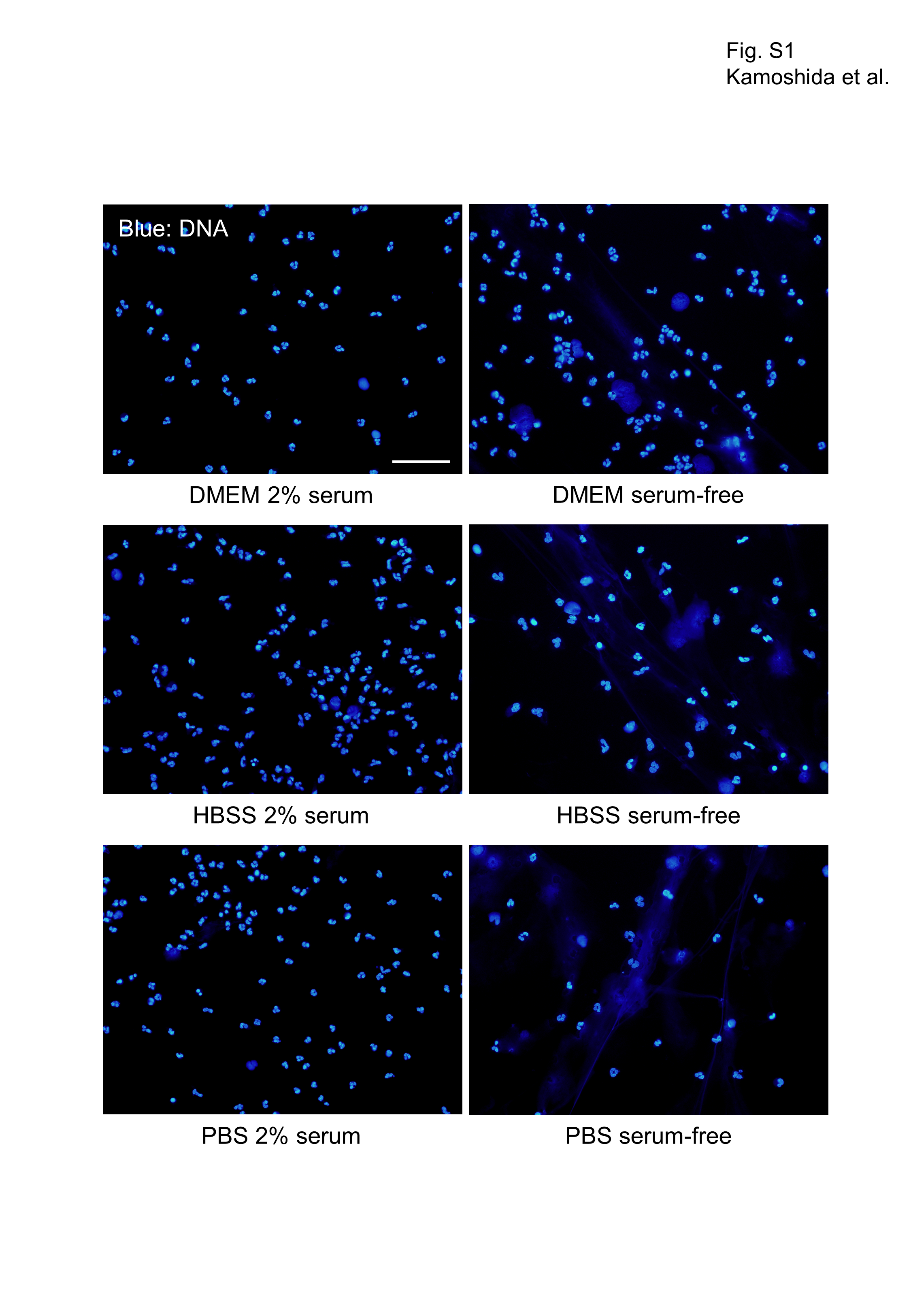

Supplement: Supplementary file 1 — Fig. S1. Effect of medium or buffer on the serum‐free induction of neutrophil extracellular traps (NETs). [file FEB4-7-877-s001.tif]
